# A surgical feasibility trial from the surgeon's perspective: a qualitative study

**DOI:** 10.1186/1745-6215-14-S1-P100

**Published:** 2013-11-29

**Authors:** Sharon McCann, Jonathan Cook

**Affiliations:** 1 Health Services Research Unit, University of Aberdeen, Aberdeen, UK

## 

Designing and conducting surgical trials is regarded as problematic, incurring a broad range of practical and methodological challenges. However, there is an increasing awareness of the need for qualitative research as part of the feasibility stage prior to the commencement of surgical trials.

We carried out semi-structured interviews with surgeons (n=11) participating in a UK single centre feasibility trial comparing single port laparoscopic surgery versus standard 3 port laparoscopic surgery for appendicectomy. We explored surgeon's reasons for agreeing to participate in the trial, and their views with regard to feasibility and acceptability issues of conducting the trial. Interviews were analysed using the ‘constant comparative' method.

Results suggested that there was variable understanding and agreement with regard to the clinical importance of what the trial was investigating. Surgeons' willingness to participate in the trial seemed strongly linked to perceptions of there being little difference in the two procedures being compared thus facilitating their readiness to participate in the trial as it potentially posed little or no perceived risk to their patients. However, it was found there was some confusion regarding the trial inclusion criteria and the term equipoise which underpinned non-entry of some potentially eligible patients. Trial feasibility issues such as financial and administrative support for surgeons was identified as important for optimising the ‘buy in' of surgeons to participate in a trial.

